# Antimicrobial and antibiofilm activities of *Casearia sylvestris* extracts from distinct Brazilian biomes against *Streptococcus mutans* and *Candida albicans*

**DOI:** 10.1186/s12906-019-2717-z

**Published:** 2019-11-12

**Authors:** Sabrina M. Ribeiro, Érick D. O. Fratucelli, Paula C. P. Bueno, Marlene Kelly V. de Castro, Amanda Alcalá Francisco, Alberto José Cavalheiro, Marlise I. Klein

**Affiliations:** 1 Department of Dental Materials and Prosthodontics, São Paulo State University (UNESP), School of Dentistry, Rua Humaitá, 1680. Araraquara, Sao Paulo, 14801-903 Brazil; 2 Department of Organic Chemistry, São Paulo State University, Rua Prof. Francisco Degni 55, Araraquara, São Paulo, 14800-060 Brazil; 3 Present address: University of São Paulo (USP), School of Pharmaceutical Sciences, Ribeirão Preto, São Paulo, Brazil

**Keywords:** *Streptococcus mutans*, *Candida albicans*, *Casearia sylvestris*, Biofilm

## Abstract

**Background:**

Dental caries is a biofilm-diet-dependent worldwide public health problem, and approaches against microorganisms in cariogenic biofilms are necessary.

**Methods:**

The antimicrobial and antibiofilm activities of 12 *Casearia sylvestris* extracts (0.50 mg/mL) from different Brazilian biomes (Atlantic Forest, Cerrado, Caatinga, Pampa, and Pantanal) and varieties (*sylvestris*, *lingua*, and intermediate) were tested against two species found in cariogenic biofilms (*Streptococcus mutans* and *Candida albicans*). The extracts effective against *S. mutans* were used to evaluate the "adhesion strength" of this bacterium to the salivary pellicle and initial glucan matrix and the *S. mutans-*GtfB activity. Also, the antimicrobial activity against *S. mutans* of three fractions (methanol, ethyl acetate, and hexane; 0.25 mg/mL) from the extracts was evaluated.

**Results:**

Three extracts from the Atlantic Forest variety *sylvestris* (FLO/SC, GUA/CE, PRE/SP) reduced ≥50% (> 3 logs) *S. mutans* viable population (*p* < 0.0001 vs. vehicle), while two extracts from the same biome and variety (PAC/CE, PRE/SP) decreased ≥50% of the viable counts of *C. albicans* (*p* < 0.0001 vs. vehicle). For *S. mutans* biofilms, three extracts (GUA/CE, PAC/CE, PRE/SP) reduced the biomass by ≥91% (*p* > 0.0001 vs. vehicle) and 100% of the microbial population (*p* < 0.0001 vs. vehicle). However, for the fungal biofilm, two extracts (PAC/CE, PRE/SP) reduced the viable counts by ≥52% (*p* < 0.0001 vs. vehicle), but none reduced biomass. The extracts with higher antimicrobial and antibiofilm activities presented higher content of clerodane-type diterpenes and lower content of glycosylated flavonoids than the less active extracts. The extracts had no effect on the removal of cells adhered to the pellicle (*p* > 0.05 vs. vehicle) while promoted the detachment of a larger number of *S. mutans* cells from GtfB-glucan matrix (*p* < 0.0031 vs. vehicle), and FLO/SC, GUA/CE and PRE/SP reduced the quantity of glucans (*p* < 0.0136 vs. vehicle). Only the ethyl acetate fractions reduced the microbial population of *S. mutans* (*p* < 0.0001 vs. vehicle), except for one (PAC/CE). Among the ethyl acetate fractions, three from var. *lingua* (two from Cerrado, and one from Cerrado/Caatinga) reduced ≥83% of the microbial population.

**Conclusions:**

*C. sylvestris* extracts from Atlantic Forest var. *sylvestris* and ethyl acetate fractions from Cerrado and Cerrado/Caatinga var. *lingua* may be used as a strategy against cariogenic microorganisms.

## Background

Dental caries is still the most prevalent oral condition associated with biofilm in the world [1–3], which harms the quality of life of millions of people [1–3]. This ubiquitous disease results from complex interactions between specific oral organisms, host factors, and diet, which promote the transition from a healthy biofilm to a pathogenic one on the surface of the teeth [4, 5]. Therefore, approaches to hinder and control the formation of pathogenic biofilms can be a strategy for the prevention of dental caries.

*Streptococcus mutans* plays a key role in the modulation and transition from non-pathogenic form to highly cariogenic biofilms [6], although additional organisms may be associated [7–9]. This species is highly acidogenic and aciduric and is the main producer of the extracellular matrix in dental biofilms [5]. What makes *S. mutans* the leading producer of exopolysaccharides is that it encodes multiple exoenzymes, mainly glycosyltransferases (Gtfs) [5, 10] that are secreted into the extracellular medium and use dietary sucrose as the substrate to synthesize glucans. Gtfs become constituents of the salivary pellicle and are also adsorbed to the surfaces of *S. mutans* and other microorganisms, such as *Candida albicans*, maintaining its enzymatic activity [5, 11, 12]. Glucan synthesis in the pellicle provides additional microbial binding sites, while the polymers on the surface of microorganisms increase the cohesion between organisms [5, 11, 12].

*C. albicans* is the fungus most commonly found on human mucosal surfaces and frequently participates in the formation of polymicrobial biofilms on biotic and abiotic surfaces [13, 14], especially in the presence of dietary sucrose [15]. This fungus has an extraordinary acidogenic capacity and acid tolerance, and its association with *S. mutans* results in increased exopolysaccharides formation, enhancing biofilm cariogenicity [16–18]. In addition, *C. albicans* produces and secretes exoenzymes capable of degrading dentin collagen under acidic conditions, contributing to biofilm virulence and cariogenicity [19, 20].

Preventing the formation of this biofilm is essential to avoid the occurrence of dental caries. Chlorhexidine (a broad-spectrum antimicrobial agent) and fluoride are considered gold standard in dentistry for biofilm treatment and caries prevention, respectively. However, chlorhexidine suppresses the buccal microbiota [21] and is not suitable for daily and continuous preventive and / or therapeutic use due to its side effects [21], while fluoride provides incomplete protection against disease and has no antimicrobial effect under the conditions clinically used [22].

Therefore, it is necessary to search for strategies to control and/or modulate cariogenic biofilms and, at the same time, do not cause toxicity to the human organism. One attractive approach would be the use and/or inclusion of bioactive agents that affect the virulence of pathogenic microorganisms without unbalancing the normal microbiota of the mouth. Consequently, there is a growing interest of researchers and industry in the development of new therapies based on natural products [23]. These products could be used to reduce biofilm pathogenicity as an adjunct of fluoride for caries prevention.

Natural products have a wide range of activities and functions and have a rich history of use in traditional medicine. The prospection of compounds extracted from plant extracts with antimicrobial properties and antibiofilm is a relevant strategy for dentistry and other areas.

*Casearia sylvestris* Swartz (Salicaceae) is a plant that is distributed in the tropical and subtropical regions of Brazil, and other countries of South America and Asia. It has a huge pharmacological and cytotoxic arsenal, anti-inflammatory, antiplasmodial, and anti-ulcer properties [24]. *C. sylvestris* (“guaçatonga”) is part of popular/traditional use in Brazil [24, 25]. This plant is cited in the “National List of Medicinal Plants of Interest to SUS” (RENISUS), which contains 71 species that could treat the diseases with a high incidence in Brazil [25]. Indigenous tribes use the macerated bark of *C. sylvestris* to treat gastrointestinal disorders (i.e., diarrhea), leprosy, snake bites, and to heal wounds [24], while decocted bark is used as an anti-inflammatory [26], and for snake bite where the bark is infused for on-site application [27]. In addition, Brazilian natives use leaves of *C. sylvestris* as tonic and antispasmodic, for fever, syphilis, herpes, and snake bites [26, 28]. The usual administration is oral, and the most common form of preparation is decoction.

However, there is little elucidation about its antimicrobial activity [29, 30], while its anti-cariogenic biofilm effect is non-existent. The chemical profile of leaf extracts of *C. sylvestris* var. *sylvestris* (from Atlantic Forest) presents a rich phytochemical composition, with abundant diterpenes, considered taxonomic markers for this genus [31] while phenolic compounds (flavonoids) predominate in var. *lingua* [32, 33].

Hence, *C. sylvestris* provides a rich source of molecules that may exhibit antimicrobial and antibiofilm properties. Therefore, the current study evaluated the antimicrobial and antibiofilm activities of *C. sylvestris* leaves extracts and fractions from distinct Brazilian biomes, belonging to varieties *lingua*, *sylvestris*, and intermediate. The extracts that presented an antibiofilm response against *S. mutans* were used to verify the effect on the adhesion of this microorganism to the salivary pellicle and the initial matrix (glucans) formed on hydroxyapatite surface.

## Methods

### *Casearia sylvestris* extracts and fractions from distinct Brazilians biomes

The plant was registered in the National System of Genetic Resource Management and Associated Traditional Knowledge (SisGen; Register #A00892A) and all collections were made with the permission of the Brazilian Institute of Environment and Renewable Natural Resources (IBAMA) through the System Authorization and Information on Biodiversity (SISBIO), which provided proof of registration (SISBIO; Register # 33429–1).

The samples were collected from three different individuals of *C. sylvestris* from 12 regions from Brazil, belonging to the biomes: Cerrado, Caatinga, Atlantic Forest, Pampa, and Pantanal, in the period between June and September 2012 and 2013. Voucher specimens of all samples were sent to Agronomic Institute of Campinas (São Paulo State, Brazil) for identity confirmation and variety assignment by Prof. Dr. Roseli B. Torres.

Depending on the availability of plant material, 10 to 20 leaves were collected, and these leaves were dehydrated at 40 °C in a circulating air oven and stored in hermetically sealed plastic bags at room temperature. Then, these samples were individually ground, and 5 g of each sample were extracted three times using 30, 15, and 10 mL portions of extractor solvent (water, ethanol, and isopropyl alcohol, in a proportion of 5:3:2 - % v/v), using an ultrasonic bath. Between each extraction cycle, these samples were centrifuged (5000 *x g,* 5 min) and then filtered. Next, these extracts were combined and lyophilized, resulting in 12 freeze-dried crude extracts (Table 1). Finally, these extracts were solubilized at 6 mg/mL with 84.15% ethanol (EtOH; Sigma-Aldrich Co. St. Louis, MO) and 15% dimethyl sulfoxide (DMSO; Sigma-Aldrich Co. St. Louis, MO).

**Table 1:** Samples of *C. sylvestris* collected in different Brazilian biomes. Personal communication by Dr. Paula Carolina Pires Bueno

Population^a^	Sample Code	Morphology^b^	Biome	Climate^c^		Sun exposition
				Temperature (°C)	Precipitation (mm)	
Araraquara/SP	ARA/SP	L	Cerrado	20.4	1352	High
São Roque de Minas/MG	SRM/MG	L	Cerrado	20.6	1390	High
Cariri/CE	CAR/CE	L	Ecotone (Cerrado/Caatinga)	25.1	1086	High
Luis Antônio/SP	LUI/SP	L	Ecotone (Cerrado/Atlantic Forest)	21.9	1508	High
Mogi-Guaçu/SP	MOG/SP	I	Ecotone (Cerrado/Atlantic Forest)	20.3	1344	High
Rio Grande/RS	RIO/RS	I	Pampa (Cerrado / wetland area)^d^	18.3	1205	High
Cáceres/MT	CAC/MT	I	Pantanal (Cerrado / wetland area)^d^	26.3	1301	High
Campinas/SP	CAM/SP	I	Cerrado (Ciliary Forest)	19.3	1315	Low
Guaramiranga/CE	GUA/CE	S	Atlantic Forest	20.9	1560	Low
Pacoti/CE	PAC/CE	S	Atlantic Forest	21.5	1524	Low
Florianópolis/SC	FLO/SC	S	Atlantic Forest	20.1	1462	Low
Presidente Venceslau/SP	PRE/SP	S	Atlantic Forest (remnant area)	21.6	1207	Low

^a^Five or six individuals sampled; ^b^Morphology based on botanical classification; ^c^Climate data corresponds to the average annual values - available at https://pt.climate-data.org/ and http://www.inmet.gov.br; ^d^ these localities have high water availability; L: var. *lingua*; S: var. *sylvestris*; I: intermediate morphology

The fractions of the crude extracts were obtained as described before [28]. Solid phase extraction cartridges (SPE) containing 1 g of a mixture of silica gel 40–63 μm, 60 Å (Merck, Germany) and activated carbon (LABSYNTH, Brazil) (500 mg of each) were prepared. The columns were preconditioned with hexane/ethyl acetate 95:5 (% v/v; both from J.T. Baker, grade HPLC) and then 150 mg of the samples were applied. Fractions were eluted with 10 mL of 95:5 (% v/v) hexane/ethyl acetate (Hex fraction), 100% ethyl acetate (AcOEt fraction) and 100% methanol (MeOH fraction), respectively. Next, the solvents were evaporated with a Speed Vac modelo SPD (Thermo Scientific), resulting in dry fractions for the tests proposed. The fractions were solubilized at 1 mg/mL with 28% EtOH (Sigma-Aldrich Co. St. Louis, MO) and 5% DMSO (Sigma-Aldrich Co. St. Louis, MO) and 1X PBS (1x *phosphate buffered saline*, pH 7.4).

### Chemical characterization of the crude extracts

The chromatographic analyses used a UHPLC-DAD (Ultimate 3000 RS, Dionex) equipped with a degasser, quaternary pump, automatic sampler, UV photodiode array detector, and oven, following the methodology of Bueno et al. [33]. The separation was achieved using a C18 analytical column (Phenomenex Kinetex 150 × 2.1 mm, 2.6 μm, 100 Å) protected by a compatible pre-column. The chromatographic conditions were: flow rate of 400 μL/min, column temperature of 35 °C, and injection volume of 2 μL. The mobile phase consisted of water (A) and acetonitrile (B) using the following linear gradient elution: 10–25% B from 0 to 15 min, 25–90% B at 35 min, maintaining 90% B until 40 min and returning to the initial conditions, 10% B over 2 min, and holding for a further 3 min for column reconditioning. Absorption spectral data were collected within 45 min from 200 to 800 nm [33].

### Microbial strains and growth conditions

The strains *S. mutans* UA159 and *C. albicans* SC5314 were grown on blood agar plates (Laborclin, Brazil) and incubated (48 h, 37 °C, 5%CO_2;_ Thermo Scientific, Waltham, USA). These two species were selected as model organisms associated with dental caries development [16]. Five to ten colonies of each microorganism were inoculated in 10 mL of culture medium tryptone-yeast extract broth [TYE: 2.5% (w/v) tryptone with 1.5% (w/v) yeast extract, pH 7.0 Difco] containing 1% glucose (w/v) (TYEg) and incubated (37 °C, 5% CO_2_). After 16 h, the starter cultures were diluted (1:20) in TYEg medium, and grown until mid-log growth phase [OD_540nm_ 0.824 ± 0.169 and 1.37E+ 09 ± 6.16E+ 08 colony forming units per milliliter (CFU/mL) for *S. mutans*; OD_540nm_ 0.967 ± 0.034 and 1.96E+ 07 ± 3.63E+ 06 CFU/mL for *C. albicans*]. The inoculum for downstream assays were prepared with a defined population of 2E+ 06 CFU/mL for both strains in TYEg for antimicrobial assessment and TYE with 1% sucrose (w/v) (TYEs) for biofilms assays.

### Antimicrobial activity assay

Screenings using 0.50 mg/mL of extracts and 0.25 mg/mL of fractions were performed on 96-well flat bottom microplates (Kasvi). The concentrations were selected because of the yield of extracts and fractions and based on the literature, which considers that experiments with amounts greater than 1 mg/mL for extracts or 0.1 mg/mL for isolated compounds should be avoided [34, 35]. At these working concentrations, the vehicle was 7% EtOH [36] and 1.25% DMSO.

A 100 μL volume of the microbial inoculum (2E+ 06 CFU/mL) prepared with TYEg was transferred to the wells in the microplates. Next, 16.67 μL each extract (or its vehicle) or 50 μL fraction (or its vehicle) were added into the microbial inoculum plus 83.33 μL (for extracts) or 50 μL (for fractions) of TYEg to achieve 1E+ 06 CFU/mL in a total volume of 200 μL, and the microplates were incubated (24 h, 37 °C, 5% CO_2_). In addition to treatments and vehicle, a microbial growth control without treatment was included; controls per treatment without microbial inoculation were also performed. Each treatment was performed at least in triplicates at four distinct experiments. After 24 h of incubation, visual inspection of the wells (turbidity: microbial growth, clear: no growth) and OD_562nm_ readings (ELISA plate reader, Biochrom Ez, Cambourne, UK) were performed. However, both visual inspection data and OD readings were inconsistent because the replicates of the same experiment and from different experiments for treatments and their controls without microbial growth presented high variability; thus, these data were not shown. Attempts to determine the turbidity of cultures with a microplate reader have been reported to have flaws when used for natural compounds (as observed by visual inspection and O.D. readings), as with some test organisms the cells agglomerated at the bottom of the well and, other organisms, the cells remained in suspension [37].

Moreover, the precipitation of compounds present in extracts can generate turbidity of the wells, hindering the interpretation of the data [37]. Thus, these two analyses may not reflect the actual antimicrobial activity of the treatments (especially extracts), since the precipitation of compounds present in extracts can generate turbidity of the wells [37]. Therefore, an aliquot of each well was used for a 10-fold serial dilution (10^0^ to 10^− 5^) in saline solution (0.89% NaCl; Synth) to determine microbial cells viability by plating blood agar plates (48 h, 37 °C and 5% CO_2_). After incubation, the colonies were counted to obtain CFU/mL and calculate log and percentage of microbial growth inhibition compared to vehicle control.

### Antibiofilm activity assays using single-species biofilm models

For antibiofilm evaluation, the single concentration of 0.50 mg/mL of each extract was also used as an initial prospecting study, and the vehicle was also 7% EtOH and 1.25% DMSO. The antibiofilm activity was determined by viable counts of the microbial population (CFU/mL) and total biomass assessment using single-species biofilm models of *S. mutans* and *C. albicans*. A 100 μL volume of the microbial inoculum (2E+ 06 CFU/mL) prepared with TYEs [38] was transferred to the wells in a flat bottom microplate. Next, 16.67 μL of extract or vehicle were added into the microbial inoculum plus 83.33 μL of TYEs to achieve 1E+ 06 CFU/mL, and the microplates were incubated (24 h, 37 °C, 5% CO_2_). As for antimicrobial assay, in addition to treatments and vehicle, a microbial growth control without treatment was included, and controls per treatment without microbial inoculation (to serve as blank controls) were also performed. Each treatment was performed at least in triplicates at four distinct experiments. After 24 h of incubation, the microplates were placed on an orbital shaker at 75 rpm for 5 min (Quimis, G816 M20, São Paulo, BR). Next, the culture medium with unbound microbial cells was removed, and remaining biofilms were washed three times, with 0.89% NaCl solution to remove non-adhered cells.

#### Biomass of treated biofilms

The washed biofilms (and wells with appropriate blank controls with treatments but without microbial inoculation) were stained with an aqueous solution of 1% violet crystal and incubated (25 °C, 35 min). Next, the wells were washed using MilliQ water and air-dried for 60–90 min. Then, the violet crystal was eluted with 99% EtOH and incubation during 5 min on the orbital shaker (37 °C, 200 rpm; Quimis, G816 M20, São Paulo, BR). Next, 150 μL of the eluted volumes were transferred to another microplate, and the OD_570nm_ was measured on an ELISA plate reader (Biochrom Ez).

#### Viable counts of the microbial population of treated biofilms

The washed biofilms (and wells with appropriate blank controls with treatments but without microbial inoculation) were removed from each well using pipette tips and 0.89% NaCl solution and transferred to microtubes. Then, an aliquot of each biofilm suspension was used for a 10-fold serial dilution (10^0^ to 10^− 5^) followed by plating on blood agar plates (48 h, 37 °C, 5% CO_2_). After incubation, the colonies were counted to obtain CFU/mL and calculate log and percentage of microbial growth inhibition by each extract, compared to vehicle control.

### Effect of selected extracts on the initial formation of the Glucan matrix (GtfB activity)

#### Purification of GtfB

The GtfB enzyme was purified from the culture supernatant of *Streptococcus milleri* KSB8, following the published methodology [39, 40]. Purification procedures were performed using buffers containing two protease inhibitors as preservatives [0.1 mM phenylmethylsulfonyl fluoride (PMSF) and 0.02% sodium azide (NaN_3_)]. GtfB was purified using a chromatography column containing hydroxyapatite beads following methodology detailed before [39]. After purification, the enzyme was checked on acrylamide gel (SDS-PAGE) and stained with silver nitrate. Aliquots of the enzyme were stored at − 80 °C until use.

#### Salivary pellicle formation

Hydroxyapatite (HA) beads (Macro-Prep Ceramic Hydroxyapatite Type I 80 μm; BioRad) were used as the surface for salivary pellicle formation, to mimic the dental enamel. These beads were coated with saliva for acquired pellicle formation (sHA), following a previous protocol [41]. Stimulated whole saliva was obtained from one healthy volunteer, diluted 1:1 with adsorption buffer (AB buffer: 50 mM KCl, 1 mM KPO_4_, 1 mM CaCl_2_, 1 mM MgCl_2_, 0.1 mM PMSF, in dd-H_2_O, pH 6.5], centrifuged (1699 *x g*, 20 min, 4 °C; Eppendorf, Centrifuge 5810R) and sterilized by filtration (0.22 μm low protein binding polyethersulfone membrane filter) [41]. The Institutional Ethical Committee approved the study (CAAE: 68161417.0.0000.5416).

Aliquots of 500 μL of saliva were added to microtubes containing pre-washed (with AB buffer containing 0.1 mM PMSF and 0.02%NaN_3_) and sterilized HA beads, followed by incubation in a homogenizer (40 min, 37 °C, 24 rpm; Fisher Scientific Nutating Mixers, model 05–450-213). Next, saliva supernatant was removed, and the beads were washed three times with AB buffer containing PMSF and NaN_3_. The sHA beads were now ready for downstream assays.

#### Effect of treatments on the initial glucan matrix formation

Samples of sHA were obtained as described above. Next, 500 μL of GtfB enzyme were added to each tube followed by incubation in a homogenizer (40 min, 37 °C, 24 rpm; Fisher Scientific Nutating Mixers) and washing three times with AB buffer (containing PMSF and NaN_3_). After, 500 μL of selected extracts (0.50 mg/mL) that showed antibiofilm activity against *S. mutans* (or controls) were added to each tube, followed by incubation in a homogenizer (30 min, 37 °C, 24 rpm; Fisher Scientific Nutating Mixers) and washing three times with AB buffer (containing PMSF and NaN_3_). Then, 500 μL of sucrose substrate (100 mmol of sucrose) were added to each tube and incubated in a homogenizer (4 h, 37 °C, 24 rpm; Fisher Scientific Nutating Mixers). To stop the synthesis of glucans and to precipitate the formed glucans, 500 μL of 99% EtOH was added, followed by incubation (18 h, − 20 °C). After that, the pellets were washed three times with 70% EtOH to remove excess sucrose not incorporated into the synthesized glucans and dried (Speed Vac Concentrator RVC 2–18 CD Plus, Christ). For solubilization of the glucans produced, 200 μL of 1 N NaOH were added to each sample, followed by incubation (2 h, 37 °C, 300 rpm; Fisher Scientific Dry-Bath-Incubator, model 980FIHSCTSUs) and centrifugation. Afterward, the 200 μL supernatant was transferred to a new tube per sample, and another aliquot of 200 μL of 1 N NaOH was added to the tubes containing the treated beads and re-incubated (2 h, 37 °C, 300 rpm; Fisher Scientific Dry-Bath-Incubator). Then, the 200 μL supernatant was transferred to the corresponding tubes to obtain a final volume of 400 μL. The resulting supernatants were used for the quantification of water insoluble glucans via phenol and sulfuric acid colorimetric assay with glucose as standard [42].

### The detachment of *S. mutans* after adhesion to salivary pellicle and glucans treated with selected extracts

The microorganisms were grown to the mid-log growth phase as described above. When cultures reached the desired OD, they were centrifuged (4000×*g* for 20 min), washed with 0.89% NaCl solution, and resuspended in the same initial volume. Cultures were sonicated with a probe to dechain (30 s, 7 watts, 3 times; QSonica, model 125, Newtown, USA). OD_540nm_ was checked to adjust the concentration to 2E+ 06 CFU/mL.

#### Adhesion of S. mutans to the salivary pellicle followed by the detachment of adhered cells

Samples of sHA were obtained as described above. After that, 500 μL of selected extracts (0.50 mg/mL) that showed antibiofilm activity against *S. mutans* (or controls) were added to each tube, followed by incubation in a homogenizer (30 min, 37 °C, 24 rpm; Fisher Scientific Nutating Mixers) and washing three times with AB buffer (containing PMSF and NaN_3_). Then, 500 μL of *S. mutans* culture (2E+ 06 CFU/mL) were added to each tube, followed by incubation (1 h, 37 °C, 24 rpm) and washing three times with AB buffer (containing PMSF and NaN_3_) to remove unbound cells. Each sample was resuspended with 1000 μL of AB buffer and sonicated with a probe to detach cells adhered to sHA (30 s, 7 watts). An aliquot of each suspension was used for a 10-fold serial dilution (10^0^ to 10^− 3^) to determine the number of viable colonies by plating on blood agar plates (48 h, 37 °C, 5% CO_2_). This evalulation verifies whether the extracts used are capable of inhibiting the adherence of *S. mutans* to the salivary pellicle, but mainly, whether the cells of the microorganism that have adhered to the treated pellicle can be removed from the surface more easily by the mechanical stimulus, thus interrupting the first stage of biofilm formation.

#### Adhesion of S. mutans to the initial glucan matrix (gsHA) followed by the detachment of adhered cells

Samples of sHA were obtained as described above. Then, 500 μL of GtfB enzyme were added to each tube, followed by incubation in a homogenizer (40 min, 37 °C, 24 rpm; Fisher Scientific Nutating Mixers) and washing three times with AB buffer (containing PMSF and NaN_3_). Afterward, 500 μL of sucrose substrate (100 mmol of sucrose) containing the treatments (or controls-at the concentration 0,5 mg/mL) were added to each tube. The samples were then incubated in a homogenizer (4 h, 37 °C, 24 rpm; Fisher Scientific Nutating Mixers). After the incubation, three washes were performed with AB buffer (with PMSF and NaN_3_) to remove the treatments and excess of sucrose not incorporated in the synthesized glucans (samples of gsHA). Subsequently, 500 μL of *S. mutans* inoculum (2E+ 06 CFU/mL) were added to each tube followed by incubation in a homogenizer (1 h, 37 °C, 24 rpm; Fisher Scientific Nutating Mixers) and washing three times with AB buffer (with PMSF and NaN_3_) to remove unbound cells. Each sample was resuspended with 1000 μL of AB buffer (with PMSF and NaN_3_) and sonicated with probe to detach cells adhered to gsHA (30 s, 7 watts). An aliquot of each suspension was used for a 10-fold serial dilution (10^0^ to 10^− 3^) to determine the number of CFU by plating on blood agar plates (48 h, 37 °C, 5%CO_2_). This evalulation verifies whether the extracts used are able to inhibit the adhesion of *S. mutans* to the initial glucan matrix, but mainly, if the cells of the microorganism that have adhered to the treated glucans can be removed by the mechanical stimulus more easily of the surface, thus interrupting the stage biofilm formation.

### Cytotoxicity of selected extracts

For cytotoxicity assays, the concentration of 0.5 mg/mL of each selected extract (PAC/CE, FLO/SC, PRE/SP, GUA/CE, MOG/SP, SRM/MG, and ARA/SP) was used.

#### Cell culture

Keratinocytes NOK-si lineage [43] cells were grown in Dulbecco’s Modified Eagle’s Medium (DMEM, GIBCO, Grand Island, NY, USA) with 2 mM glutamine; containing 10% fetal bovine serum (FBS, GIBCO, Grand Island, NY), penicillin G (10,000 μg/mL), streptomycin (10,000 μg/mL) and amphotericin (25 μg/mL) (Invitrogen). The culture was incubated at 37 °C and an atmosphere of 5% CO_2_. The cells were grown to confluency (90%), washed with 1X PBS phosphate buffer (140 mM NaCl, 3.0 mM KCl, 4.30 mM Na_2_HPO_4_, 1.40 mM KH_2_PO_4_), removed with trypsin (0.05%) / EDTA solution (0.53 mM) (Invitrogen), and then centrifuged at 400 *x g* for 5 min. The cells were resuspended in the same culture medium and replated. The medium was changed every two or 3 days. For the experiments, cells between the 3rd and 8th passage were used. Cells were counted in Neubauer’s chamber and plated in 96-well microplate wells (2.0E+ 04 cells per well). The plates were incubated for 24 h (5% CO_2_; 37 °C).

#### Cell viability assay – MTT

Cytotoxicity resulting from the presence of treatments (0.50 mg/mL of extracts, vehicle control, and death control—0.11% Triton X-100) and untreated control (cell viability control) on monolayer cells was determined by the colorimetric assay of cell viability MTT [3- (4, 5-dimethylthiazol-2-yl) 2, 5-diphenyltetrazolium bromide] (Sigma) [44, 45].

This assay was performed using cell culture in monolayer, 1 h after contact with extracts and controls included in the culture medium. After the incubation period, the cells were washed with 500 μL of PBS (pH 7.4) and incubated (4 h; 37 °C; 5% CO_2_) with 250 μL of MTT solution (5.0 mg/mL). Then the forming crystals were solubilized in 250 μL of 2-propanol added to each well. Spectrophotometric measurements were performed at a wavelength of 562 nm. Two experimental occasions were performed with 4 replicates per occasion (*n* = 8). The data obtained were converted into a percentage of viable cells and compared to the control without treatment (control of cell viability). The ISO 10993-5:2009 guideline was used to determine the cytotoxicity level [46].

### Statistical analyses

Statistical analyses were performed using GraphPad Prism7 software (GraphPad Software, Inc., La Jolla, CA, USA), with a 5% significance level. The Shapiro-Wilk normality test was used. Most data presented a non-normal distribution and were evaluated by the Kruskal-Wallis non-parametric test, followed by Dunn’s multiple comparison test. Only the fractions results presented normal distribution and were assessed by two-way ANOVA considering the factors biome and fractions, followed by Tukey’s multiple comparisons test.

## Results

### Characteristics of *C. sylvestris* extracts from distinct Brazilian biomes

In an earlier study, an analytical method was developed using liquid chromatography and chemometrics to evaluate and differentiate two varieties of *C. sylvestris Swartz* (Salicaceae) from the State of São Paulo (Brazil) based on secondary metabolite profiles [33]. Previously, the analytical studies related only to the analysis of clerodane-type diterpenes. Afterward, this method was also applied to describe the flavonoid composition of *C. sylvestris* varieties [47] and the analysis of the composition of leaves extracts of other Brazilian states. Thus, considering the importance of the inclusion of phenolic compounds in analyses, a strategy was employed to simultaneously extract and detect the largest number of compounds of both chemical classes. Two peaks were selected from the resulting chromatograms and included both phenolic compounds (more precisely, glycosylated flavonoids, detected at 254 nm) and clerodane-type diterpenes (detected at 235 nm) [33]. This new strategy allowed an exhaustive chromatographic analysis of *C. sylvestris* and the results showed that the varieties presented an interesting distribution according to their original ecosystems, suggesting a strong correlation with the main metabolites found in each group of species. In addition to the inherent morphological differences, it was also possible to observe differences in the composition of the secondary metabolite within each group, depending on the place where the samples were collected [33].

**Fig. 1:**
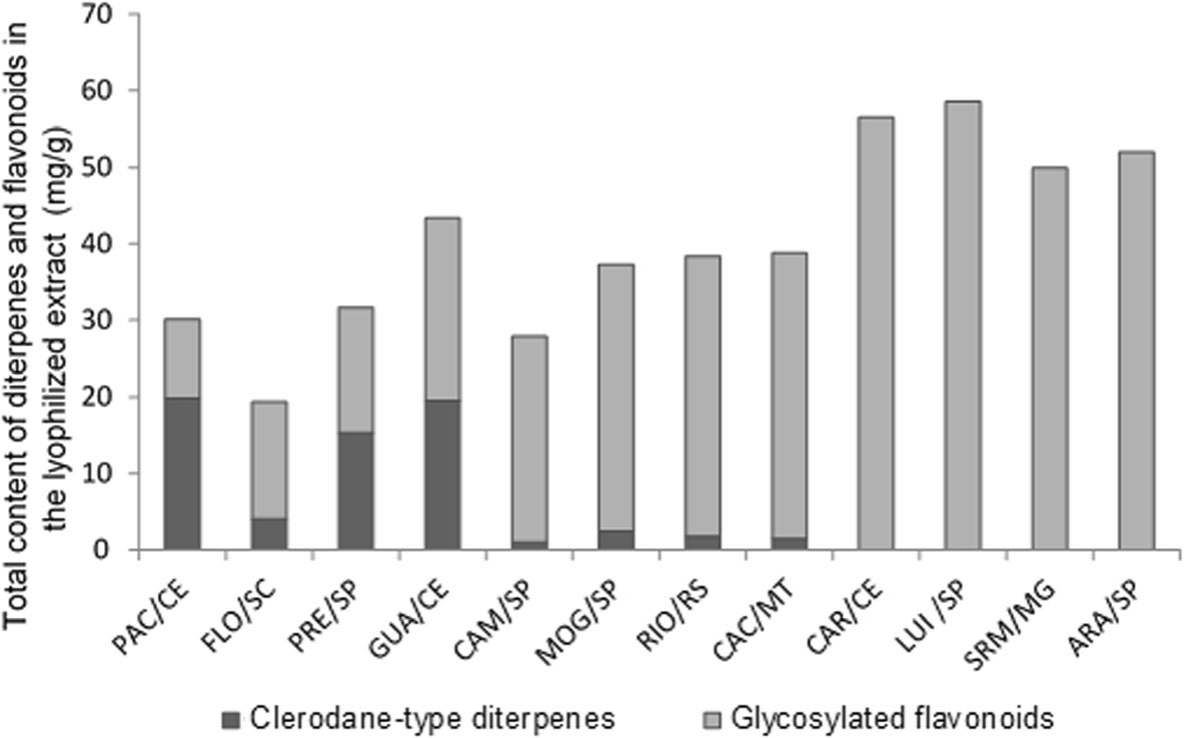
Quantity of clerodane-type diterpenes and glycosylated flavonoids in *C. sylvestris* extracts from Brazilian biomes. The letters *S*, I and *L* indicate the varieties *sylvestris*, intermediate and *lingua*, respectively. (Personal communication by Dr. Paula Carolina Pires Bueno)

Here, extracts from *C. sylvestris* var. *sylvestris* (PAC/CE, FLO/SC, PRE/SP, and GUA/CE) presented higher content of clerodane-type diterpenes and lower content of glycosylated flavonoids. However, the extracts of intermediate morphology (CAM/SP, MOG/SP, RIO/RS, and CAC/MT) present higher content of glycosylated flavonoids and lower content of clerodane-type diterpenes, while extracts from var. *lingua* (CAR/CE, LUI/SP, SRM/MG, and ARA/SP) present the highest content of glycosylated flavonoids (Fig. 1). Thus, not only the biome but the morphology interfered with the composition of tested extracts.

### Antimicrobial activity

**Fig. 2:**
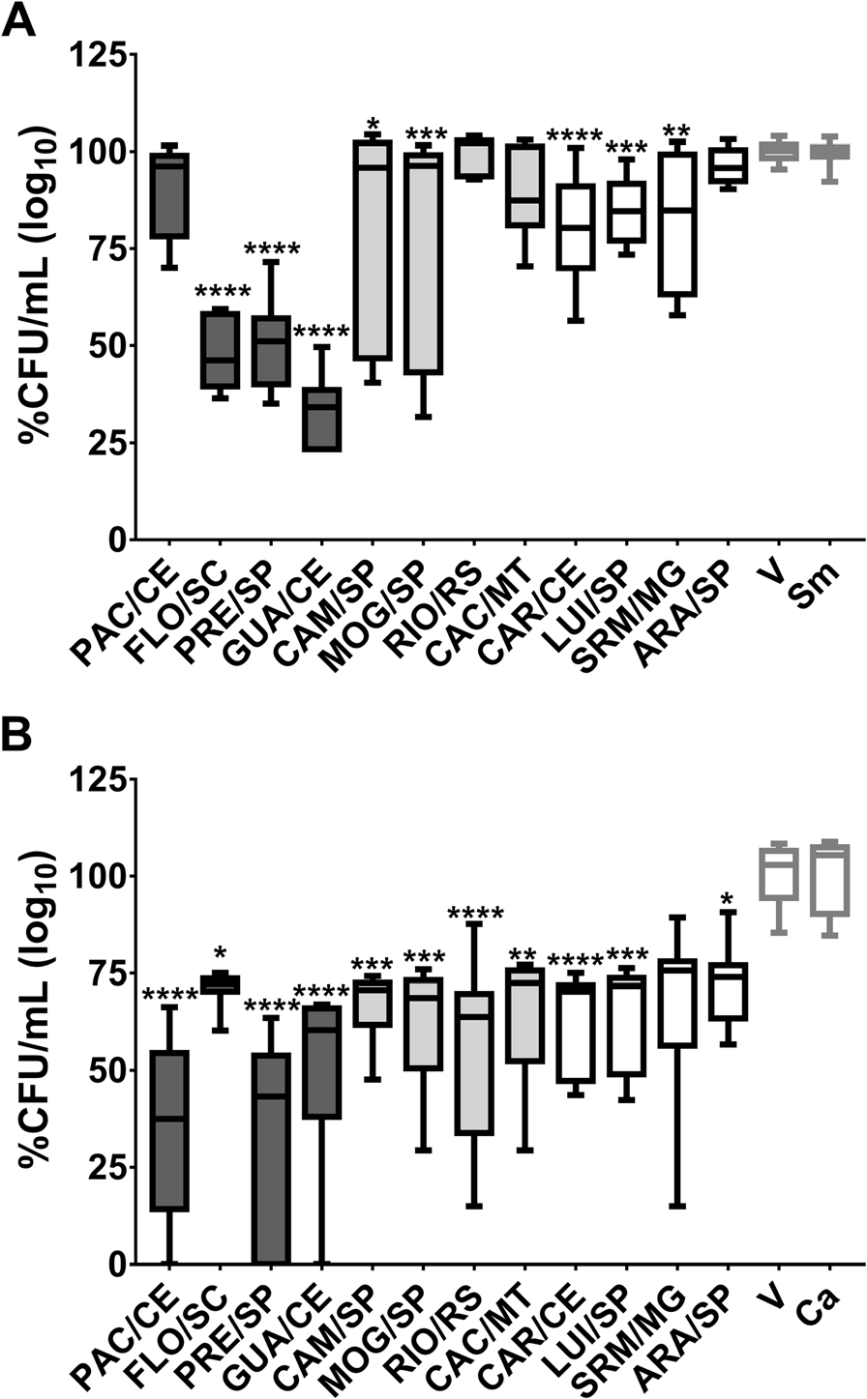
Antimicrobial activity of *C. sylvestris* extracts from Brazilian biomes against *S. mutans* and *C. albicans*. **a** shows *S. mutans* data while (**b**) depicts *C. albicans* data. The data are median (traces) and interquartis (boxes) for the 12 extracts evaluated. The error bars represent the maximum and minimum values. Asterisks denote statistically significant difference of a specific extract versus vehicle (V) control, where: ****p ≤ 0.0001; ***p ≤ 0.001; ** ≤ *p* < 0.01; and *p ≤ 0.05 (Kruskal-Wallis test, followed by Dunn’s multiple comparisons test). Each species growth control (without treatment) is represented as Sm for *S. mutans* and Ca for *C. albicans.* The colors of the bars in each graph represent the variety to which the extracts belong, being, in dark gray color: var. *sylvestris*; light gray: var. intermediate and white: var*. lingua*

The CFU/mL data were converted to log_10_ percentages to evaluate which extracts at the concentration tested inhibited > 50% of the viable counts of microbial population per tested strains (IC_50_ or > 3 logs) [48]. Significant reductions in the microbial population of *S. mutans* were seen when treated by FLO/SC (53.84% reduction), GUA/CE (65.91% reduction) and PRE/SP (48.93%) compared with the control vehicle (*p* < 0.0001, Fig. 2a). There was also a significant reduction in the microbial population of *C. albicans* when treated with PAC/CE (50.3% reduction), and PRE/SP (52.98% reduction) (*p* = 0.0001 vs. vehicle; Fig. 2b); these extracts belong to var. *sylvestris* and contemplate the Atlantic Forest biome.

**Fig. 3:**
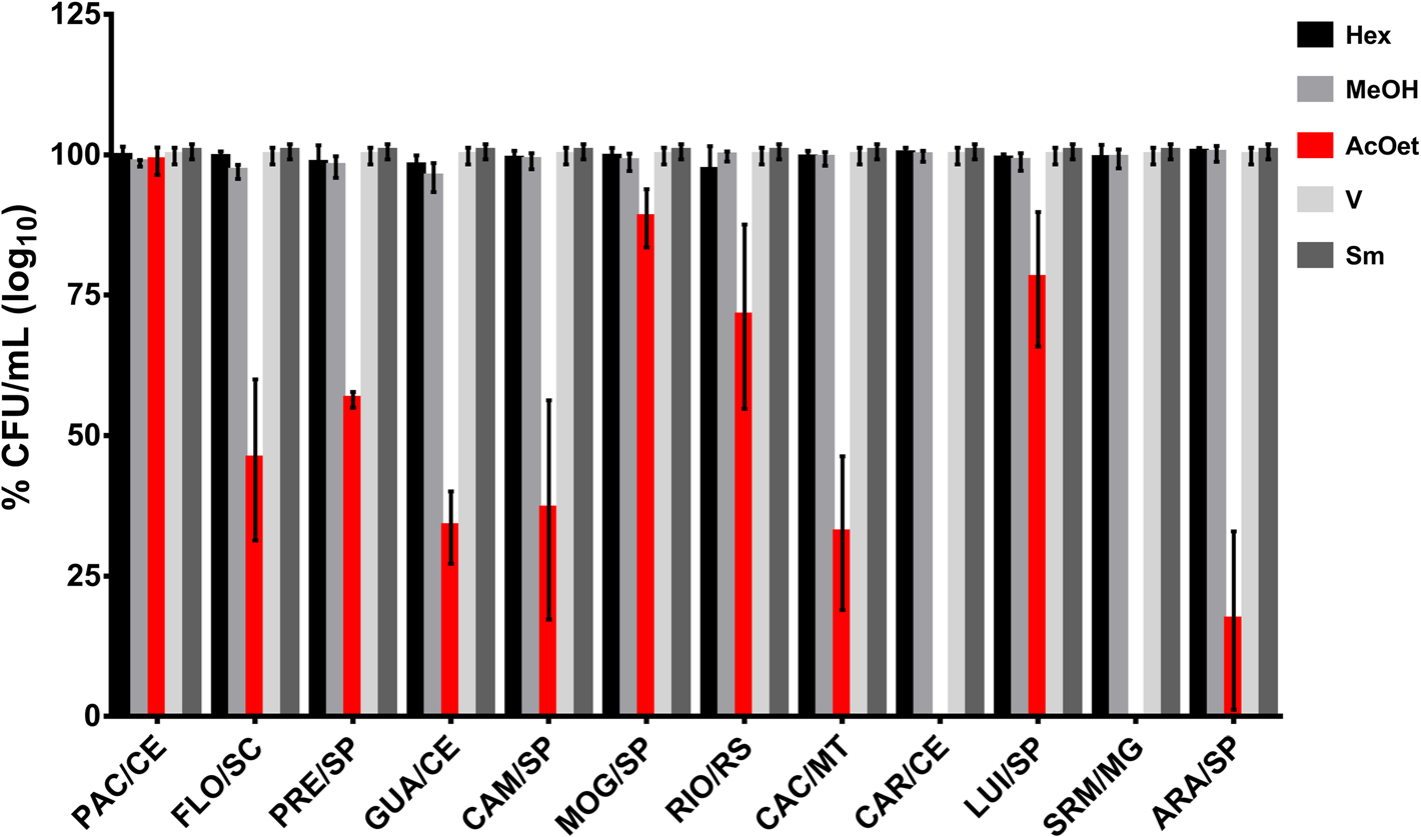
Antimicrobial activity of three distinct fractions *C. sylvestris* from distinct Brazilian biomes against *S. mutans*. Data depicted are average and standard deviation. There was statistically significant difference between fraction AcOEt versus vehicle (V) control for all, except PAC/CE (*p* ≤ 0.0001; two-way ANOVA between the bioma and fraction factors showed that the interaction between the factors is significant by factor *p* < 0.0001; biome *p* < 0.0001 and fraction *p* < 0.0001, followed by the Tukey multiple comparisons test; *p* > 0.050)

The antimicrobial activity against *S. mutans* was also evaluated for the 36 fractions of the extracts, being MeOH, AcOEt, and Hex fractions. The AcOEt fractions of all extracts reduced the microbial population of *S. mutans* (*p* < 0.0001 vs. vehicle), except PAC/CE (*p* > 0.05 vs. vehicle). The AcOEt fractions ARA/SP, CAR/CE, and SRM/MG caused a greater reduction in the viability of the *S. mutans* population (82.91, 92.76, and 100%, respectively) compared to the other AcOEt fractions (Fig. 3). The extracts to which these fractions belong are var*. lingua* and Cerrado and Ecotone (Cerrado/Caatinga) biomes and express a high content of glycosylated flavonoids. However, AcOEt is a solvent with optimum polarity to extract the diterpenes, especially the casearins, while the flavonoids are retained in the extraction cartridge. Thus, the resulting fraction has a higher content of diterpenes of the type clerodane compared to glycosylated flavonoids (Bueno PCP, unpublished observation).

### Antibiofilm activity

**Fig. 4:**
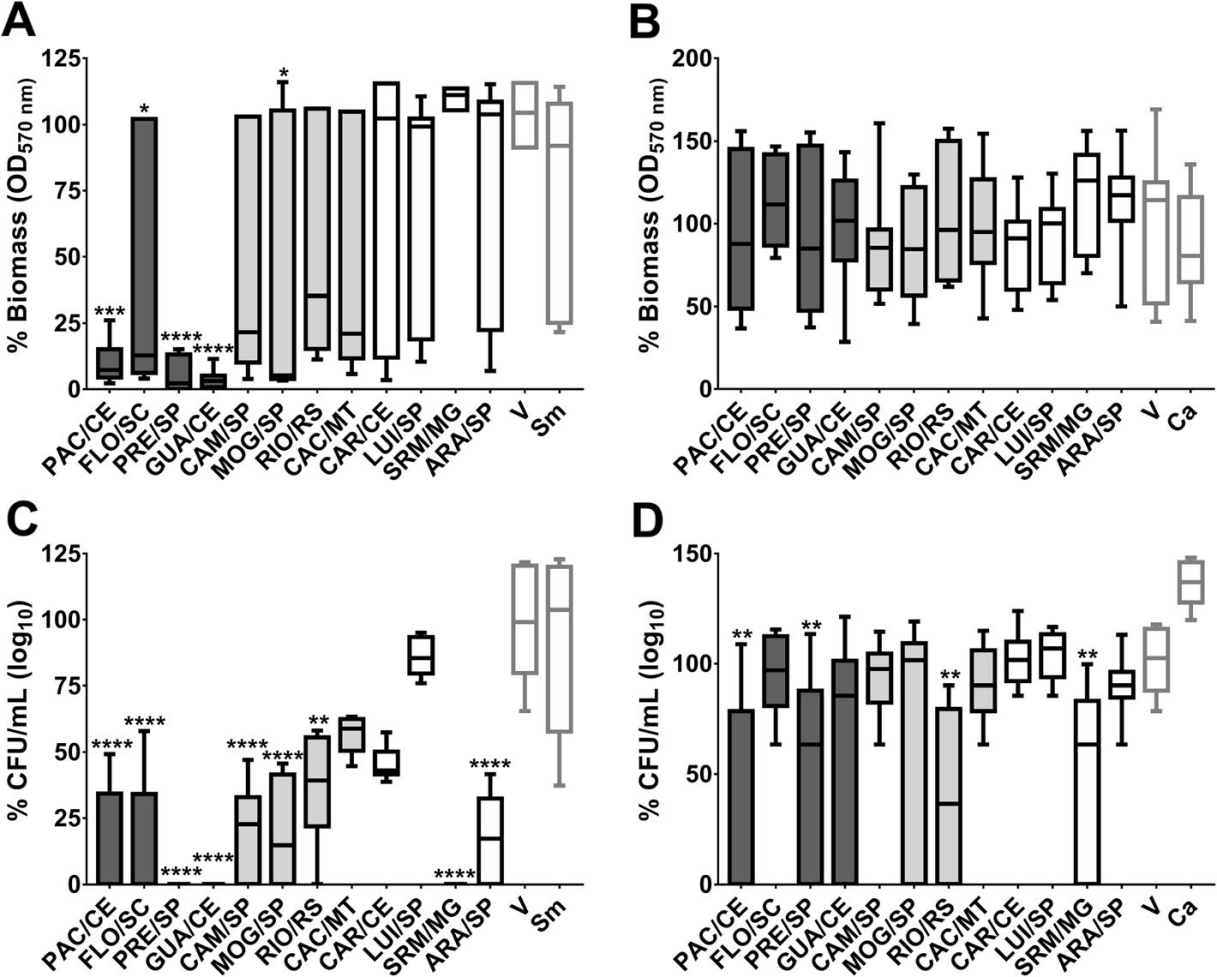
Antibiofilm activity of *C. sylvestris* extracts from Brazilian biomes against *S. mutans* and *C. albicans*. The biomass data obtained via violet crystal method are shown in (**a**) and (**b**) for *S. mutans* and *C. albicans*, respectively. The microbial viable counts data are shown in (**c**) and (**d**) for *S. mutans* and *C. albicans*, respectively. Note that the extracts presented more effect against *S. mutans* biofilms than against *C. albicans* ones. The data described are median (traces) and interquartis (boxes) of the 12 tested extracts. The error bars represent the maximum and minimum values.Asterisks denote statistically significant difference of a specific extract versus vehicle (V) control, where: ****p ≤ 0.0001; ***p ≤ 0.001; ** ≤ *p* < 0.01; and *p ≤ 0.05 (Kruskal-Wallis test, followed by Dunn’s multiple comparisons test). Each species growth control is represented as Sm for *S. mutans* and Ca for *C. albicans*. The colors of the bars of the graph represent the variety to which the extracts belong, being, in dark gray color: var. *sylvestris*; light gray: var. intermediate and white: var*. lingua*

Biofilm data were converted into percentages and extracts that reduced biofilm biomass and/or counts of viable microorganisms averaged more than 50% were considered promising for testing against specific virulence mechanisms. When compared to vehicle control, the highest biomass reduction of *S. mutans* was observed in biofilms treated with the extracts GUA/CE (95.87%), PAC/CE (91%), and PRE/SP (99.76%) (*p* < 0.0001; Fig. 4a). In addition, significant reductions in the viable count of the bacterial population were observed for ARA/SP, CAM/SP, FLO/SC, GUA/CE, MOG/SP, PAC/CE, PRE/SP, and SRM/MG (p < 0.0001 vs. V; Fig. 4c). Biofilms treated with GUA/CE, PAC/CE, PRE/SP, and SRM showed a 100% median reduction in *S. mutans* viable counts (Fig. 4c).

The biofilms of *C. albicans* treated with PAC/CE, PRE/SP, RIO/RS, and SRM/MG showed a reduction of the viable counts of the fungus (*p* < 0.0095 vs. vehicle; Fig. 4d), but for RIO/RS and SRM/MG there was high variability of the obtained data. The biomass data also presented high variability, and no differences between treatments with extracts and vehicle control were identified (Fig. 4b).

### Effect of selected extracts on GtfB activity

**Fig. 5:**
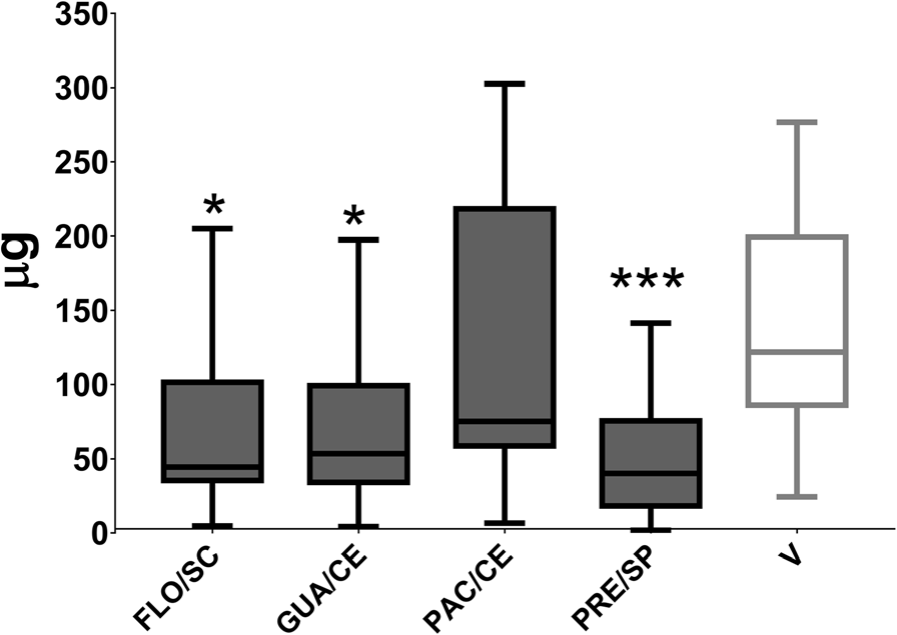
Effect of FLO/SC, GUA/CE, PAC/CE, and PRE/SP extracts on GtfB activity. The data described are median (traces) and interquartis (boxes). The error bars represent the maximum and minimum values. Asterisks indicate statistically significant differences from a specific extract versus vehicle control (V), where: ** *p* < 0.0136 and *** *p* = 0.0002 (Kruskal-Wallis test, followed by Dunn’s multiple comparison test). Only the PAC / CE extract did not affect GtfB activity (*p* = 0.6032). The colors of the bars of the graph represent the variety to which the extracts belong, being, in dark gray color: var. *sylvestris*

The effect of extracts on GtfB activity of *S. mutans* was evaluated for treatments with FLO/SC, GUA/CE, PAC/CE, and PRE/SP extracts, as these extracts presented better results for antimicrobial and antibiofilm analyses. Data from 3 experiments in triplicate, with three replicate readings are shown in Fig. 5. The FLO/SC, GUA/CE, and PRE/CE extracts reduced the amount of glucans formed by GtfB (*p* < 0.0136 vs. V), however, PAC/CE did not affect the amount of glucans (*p* = 0.6032).

### The detachment of *S. mutans* after adhesion to salivary pellicle and to the initial matrix of glucans treated with selected extracts

**Fig. 6:**
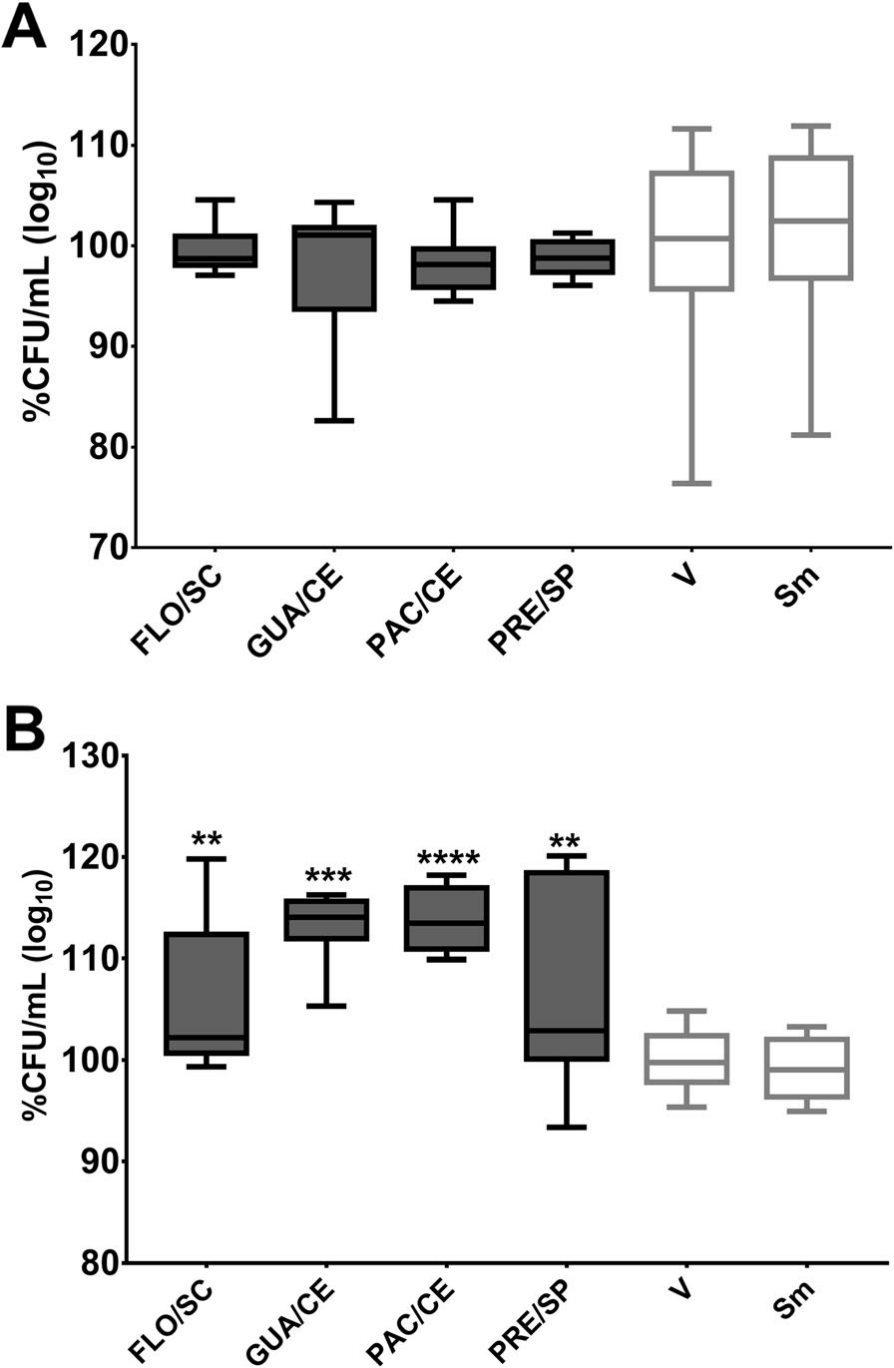
*S. mutans* detachment after adhesion to the treated salivary pellicle and initial matrix of glucans. Post-release data of *S. mutans* to the treated salivary pellicle and glucans are shown in (**a**) and (**b**), respectively. There was no difference between the control vehicle (V), and the extracts tested for both analyses. The percentage of CFU/mL was obtained considering the vehicle control (V) as 100%. The data described are median (traces) and interquartis (boxes). The error bars represent the maximum and minimum values. The asterisks denote a statistically significant difference of a specific extract versus vehicle control (V), where ****p = 0.0001 and **p < 0.0031 (Kruskal-Wallis test, followed by the multiple comparison test of Dunn). The growth control is represented by Sm for S. mutans. The colors of the bars of the graph represent the variety to which the extracts belong, being the color dark gray to var. *sylvestris*

The quantification of *S. mutans* cells that were detached by mechanical stimulation after adhesion to the salivary pellicle and glucans was evaluated for treatments with the extracts FLO/SC, GUA/CE, PAC/CE, and PRE/SP, as these four extracts presented better results for antimicrobial and antibiofilm evaluations. No extract interfered with the removal of cells adhered to the treated pellicle (*p* > 0.05 vs. vehicle; Fig. 6a); thus, these extracts may not modify the pellicle per se. However, a greater number of *S. mutans* cells were detached after adhesion to the treated glucan matrix (*p* < 0.0031 vs. vehicle; Fig. 6b).

### Cytotoxicity of selected extracts

**Fig. 7:**
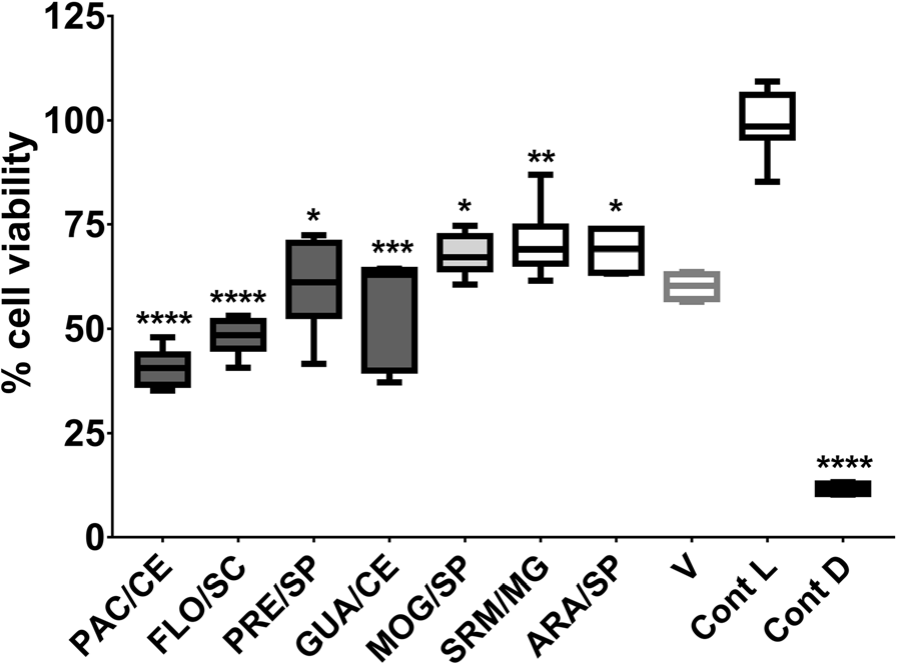
Cell viability of keratinocytes NOK-si after exposure to selected extracts. The data described are the medians and the interquartile ranges. The data described are median (traces) and interquartis (boxes). The error bars represent the maximum and minimum values. “Cont L” indicates cell viability control and “Cont D”, the cell death control. The percentage of cell viability was obtained considering the cell viability control as 100%. The asterisks denote a statistically significant difference of a specific extract versus cell viability control control (Cont L), where ****p < 0.0001, ***p = 0.0004, **p = 0.0088, and *p ≤ 0.0204 (Kruskal-Wallis test, followed by the multiple comparison test of Dunn). The colors of the bars of the graph represent the variety of the extracts, being, in dark gray: var. *sylvestris*; in light gray: var. intermediate; and blank: var. *lingua*

The cell viability data (NOK-si lineage) showed that exposure of these cells for 1 h to the tested extracts and the vehicle-control caused cell death, as compared to the viability control (Fig. 7). The ISO 10993-5:2009 provides guideline for the cytotoxicity classification as not cytotoxic, when there is inhibition of cell viability of less than 25% compared to the control group (cell viability control-“Cont L”); slightly cytotoxic, with inhibition between 25 and 50% in comparison with the control group (“Cont L”); moderately cytotoxic, with inhibition between 50 and 75% in comparison with the control group (“Cont L”); and strongly cytotoxic, with inhibition higher than 75% of that of the control group (“Cont L”) [46]. Therefore, the treatments promoted slight cytotoxicity (PRE/SP, GUA/CE, MOG/SP, SRM/MG, ARA/SP, and V control) and moderate cytotoxicity (PAC/CE and FLO/SC). However, there was no difference between extracts and vehicle control (*p* > 0.9999).

## Discussion

The results obtained in this study indicate that certain extracts and fractions of *C. sylvestris* exhibit potent antimicrobial and antibiofilm effects against two of the main microorganisms related to the pathogenesis of dental caries. Previous studies that performed systematic screenings of *C. sylvestris* demonstrated a varied phytochemical composition for leaf extracts [32, 33]. This varied phytochemical composition (presence of clerodan diterpenes and glycosylated flavonoids) provides a rich source of molecules that may exhibit cariostatic properties, and their potential must be explored. Also, the natural origin of these extracts allows them to be more easily accepted for the long-term control of biofilm-mediated diseases, such as dental caries, in addition to being less costly [49].

Here, the tested samples of *C. sylvestris* extracts and fractions were from three distinct varieties (*lingua*, intermediate, and *sylvestris*) that cover the five Brazilian biomes. The relevance of screening samples of different varieties and biomes is due to the variability of chemical composition concerning secondary metabolites. The chemical composition of *C. sylvestris* is related and/or conditioned by the biomes, and mainly associated with the respective predominant morphotypes and, therefore, under strong genetic control. The different origin biomes influence the production of secondary metabolites in the *C. sylvestris* varieties and, therefore, also associated with the respective predominant morphotypes. There was evidence of the differential production of flavonoids and clerodanic diterpenes by the var. *lingua* and *sylvestris*, respectively, which was constant throughout the circadian cycle and had modulations only in the reproductive period (Bueno PCP, unpublished observation). This variability should, therefore, be considered for the biological activity of natural products derived from *C. sylvestris*.

Although Minimum Inhibitory Concentration (MIC) and Minimum Bactericidal and Fungicidal Concentration (MBC/MFC) tests are widely used to determine the activity of new compounds [37, 48, 50], the number of extracts and fractions evaluated here has limited the use of that methodology. Therefore, based on concentrations reported in the literature [34, 35], an antimicrobial activity screening was performed using a single concentration of each extract. This screening model allowed a larger number of samples to be evaluated in a single experiment and identified extracts/fractions with potential inhibitory effects. Nevertheless, the antimicrobial assay data obtained showed that the concentration of 0.50 mg/mL of specific extracts inhibited > 50% of the viable counts of the microbial population for both the bacteria and the fungus (IC_50_ or > 3 logs) [48]. For *S. mutans*, the extracts that inhibit > 3 logs were FLO/SC, GUA/CE, and PRE/SP; while both PAC/CE and PRE/SP reduced > 3 logs of *C. albicans* population. Therefore, the four extracts FLO/SC, GUA/CE, PAC/CE, and PRE/SP could be used to obtain the MIC and MBC data in a future study. Similarly, among the 12 AcOEt fractions at 0.25 mg/mL, seven decreased > 3 logs of *S. mutans* (FLO/SC, GUA/CE, CAM/SP, CAC/MT, CAR/CE, SRM/MG, and ARA/SP), and could also be used for MIC evaluation in the future. Thus, the current findings will direct studies for optimization of extract/fraction concentration (or extract/fraction combination) in more complex in vitro (e.g., microcosm) and in vivo models.

The single-species biofilm model used in this study has been recommended for initial screenings of new anti-caries agents [51] and, although it does not mimic the complexity of the microbiota of the teeth' coronal surfaces, expresses a critical virulence characteristic of the biofilm, the polysaccharide matrix [52]. The advantages of this model include high reproducibility of biofilm formation; allows high throughput screening of multiple compounds and concentrations in a single experiment [38, 53]; the treatment steps can be consistently controlled [54, 55].

In addition, biofilms using a single microorganism are advantageous for the analysis of the mechanisms of action of therapeutic agents, especially in the glucan-mediated processes involved in the formation of the polysaccharide matrix in the *S. mutans* biofilm [52]. Single-species model is also resourceful for the formation of hyphae, a critical virulence factor of *C. albicans*. This fungus has also been chosen for the initial screening of extracts as it is one of the microorganisms frequently detected in biofilms (dental plaque) of children affected by early childhood caries [56] and provides an increase in the binding sites of *S. mutans* derived Gtfs [57]. The results of antimicrobial and antibiofilm tests (biomass and viable population data) indicate that FLO/SC, GUA/CE, PAC/CE, and PRE/SP made the most significant reductions for *S. mutans*. These four extracts are from the Atlantic Forest, var. *sylvestris*.

Among these extracts, PAC/CE and PRE/CE presented a concomitant reduction of *C. albicans* viable counts in both antimicrobial and antibiofilm evaluations. These findings can be attributed to the extracts’ phytochemical composition, which is marked by the simultaneous presence of phenolic compounds (glycosylated flavonoids) and clerodane-type diterpenes. The presence of both plant metabolites in these extracts can be particularly advantageous over the other extracts because flavonoids and terpenoids belong to the classes of compounds reported to be effective for controlling virulence factors of cariogenic microorganisms [58]. Thus, the best biological activity observed for the four extracts (FLO/SC, GUA/CE, PAC/CE, and PRE/SP) can be attributed to a potential synergism between the plant metabolites, which promotes multi-target effects. This hypothesis is because the other extracts evaluated present in their composition the predominance of only one of the metabolite classes, and none of them produced significant inhibitory effects.

The current study aimed not to isolate bioactive compounds from extracts and fractions and to identify their possible targets of action, but to perform a systematic screening to identify which ones have a potential biological activity to control/prevent cariogenic biofilm. However, knowledge of the secondary metabolites found in *C. sylvestris* leaf extracts provided the basis for the interpretation of the results found. *C. sylvestris* has secondary metabolites that confer different pharmacological properties to the plant and justify its use in folk medicine. A previous phytochemical study provided valuable information about the phenolic components from the leaves of the ARA/SP extract, following the same extraction method as the extracts tested here [33, 47]. Fourteen glycosylated flavonoids and one catechin were isolated and identified, as follows: (+)-catechin, quercetin-3-O-α-L-rhamnopyranosyl-(1 → 6)-ß-D glucopyranoside (rutin), isorhamnetin-3-O-α-L-rhamnopyranosyl-(1 → 2)-ß-Dglucopyranoside (isorhamnetin-3-O-neo-hesperidoside, isorhamnetin-3-O-α-Lrhamnopyranosyl-(1 → 6)-ß -D-glucopyranoside (narcissin) e isorhamnetin-3-O-α-Lrhamnopyranosyl-(1 → 2)-α -L-arabinopyranoside [47]. A previous study [33] with ARA/SP, CAM/SP, MOG/SP, and PRE/SP samples selected two peaks from the chromatograms and included the clerodane-type diterpenes (casearins) detected at 235 nm. Other chemical investigations have isolated 41 clerodane-type diterpenes, including casearins [59–64] and casearvestrins [65].

Flavonoids have different pharmacological properties, including antimicrobial and antioxidant activities [66, 67]. These compounds may be complexed with extracellular and soluble proteins as well as with bacterial cell wall and can also inhibit Gtfs activity [66, 67]. In contrast, terpenoids can promote rupture of the microbial cell membrane by their lipophilic characteristics [68]. Therefore, the activity of these compounds may justify the antimicrobial activity against *S. mutans* (≥50% reduction of viable bacterial counts) caused by Atlantic Forest extracts, var*. sylvetris*. Further investigation is needed to corroborate these effects of *C. sylvestris*.

The *S. mutans* viable counts data after treatment with ARA/SP, CAR/CE, and SRM/MG ethyl acetate fractions confirm a significant reduction in the bacterium viable population ≥ 80%, in contrast to the other fractions. The fractions were fractionated from extracts with a higher content of glycosylated flavonoids. Although ethyl acetate fractions are fractionated from extracts with higher content of glycosylated flavonoids, the extractive method used results in fractions with higher content of diterpenes in relation to flavonoids, since ethyl acetate is a good solvent to extract clerodan diterpenes (specifically casearins) due to their polarity, so even in extracts with low diterpene content, the content that is present will be extracted, varying only the yield between each resulting fraction (Bueno PCP, unpublished observation). The terpenes may affect the virulence and could be responsible for the bactericidal effect found [67, 69, 70]. Thus, they should now be considered for future studies concerning activity against oral biofilms and anti-Gtfs activity, in addition to the paramount importance of isolating and identifying bioactive compounds from fractions.

Among the mapped phenolic compounds identified in ARA/SP [33], two of them (catechins and quercetins) affect the activity of *S. mutans* Gtf enzymes [71], interfering with the synthesis of soluble and insoluble glucans in biofilms [72, 73]. Moreover, quercetin has inhibitory activity against bacterial acid production [74, 75]. This anti-Gtfs action described for the phenols also found in *C. sylvestris* may be indirectly responsible for the biomass decrease observed for treated *S. mutans* biofilms. Here, the extracts with promising antimicrobial and antibiofilm activities against *S. mutans* also inhibited GtfB activity. The anti-Gtfs effect may reduce the quantity of exopolysaccharides and may cause both the reduction of the overall biofilm volume and affect bacterial biomass, as observed here. This cascade of events would limit the binding sites available for adhesion and bacterial co-aggregation, interfering in the initial stage of biofilm formation. Another effect on Gtfs activity would be the modification of glucans produced, in which the type of glycosidic linkage could be affected. Changes in the type of and proportions of glycosidic linkages and ramifications can hinder biofilm build-up by weakening the binding of microbial cells and the overall tridimensional structure of biofilms [5].

The simultaneous presence of flavonoids and diterpenes compounds in FLO/SC, GUA/SP, PAC/CE, and PRE/SP can be particularly advantageous concerning the other extracts, as previous studies show that phenols and terpenes are effective against *S. mutans* [58, 60, 61]. Thus, here, FLO/SC, GUA/CE, and PRE/SP affected the activity of GtfB and caused a decrease in the amount of glucans produced. However, FLO/SC, PAC/CE, and PRE/SP extracts may have also affected the quality of glucans formed by GtfB, since a larger number of *S. mutans* cells were removed after adhesion to glucans when these extracts were present during their synthesis. Therefore, the four extracts from the Atlantic Forest, var. *sylvestris* could modify the glucans formed on the sHA surfaces, and this modification could weaken the adhesion of *S. mutans* to the initial glucan matrix. Insoluble glucans are virulence markers of cariogenic biofilms [5, 56]; thus, interfering with this trait is an approach to prevent dental caries. This behavior is clinically desirable because it could facilitate the mechanical removal of cells when using a formulation with these extracts in the future. In contrast, these four extracts did not modify the salivary pellicle, since no extract significantly affected the removal of cells adhered to the salivary pellicle.

Clerodane-type diterpenes are secondary metabolites of the terpene class, a class to which the diterpenes of the extracts belong. This class of compounds has been identified as responsible for the activity on *C. albicans*. Terpenes act on the permeability of fungal cells causing changes in membrane properties and their functions [76–79], cell morphology, and inhibit the growth of this fungus. Tannins (phenolic substance) are bioactive compounds of *C. sylvestris* [80] that inhibited in vitro yeast growth and inactivated the fungal cell membrane by precipitating proteins [81]. These bioactive compounds may be related to the results obtained after treatments of *C. albicans* with PAC/CE and PRE/SP. In addition, although RIO/RS and SRM/MG showed a statistically significant reduction in the biofilm population of *C. albicans*, how far this reduction is biologically significant needs to be investigated because of the data high variability. Both RIO/RS and SRM/MG presented predominantly glycosylated flavonoids, suggesting that these phenolic compound act better when in association with diterpene compounds in the extract, as found for PAC/CE and PRE/SP, probably due to an action of some component(s) that increases the stability or bioavailability of the other or increasing its metabolism [82]. Moreover, the lack of effect of extracts on biomass reduction of *C. albicans* biofilms can be attributed to the morphological difference between the tested microorganisms, since one is a Gram-positive bacterium while the other is a fungus.

Here, extracts of *C. sylvestris* were more active against *S. mutans* compared to the effects against *C. albicans*, both in antimicrobial and antibiofilm analyses. *C. albicans* has several virulence factors, such as polymorphism [influenced by quorum sensing signaling molecules tyrosol, a phenylethanoid (yeast to hyphae) and farnesol, a sesquiterpene (hyphae to yeast)], biofilm formation (presence of extracellular matrix), and control of nutrient competition [83, 84]. These are the main factors associated with resistance and drug tolerance and may cause a less pronounced effect of the extracts for this microorganism. In addition, the biomass of *C. albicans* after the treatments showed high variability and this behavior was not reported in previous studies, but it could be related both to the virulence factors of the microorganism and to the variability of the antifungal activity within the same extract [84].

The extracts PRE/SP, GUA/CE, MOG/SP, SRM/MG, ARA/SP, and V control inhibited about 25% of cell viability, thus, exerting slight cytotoxicity to the oral keratinocytes. However, two extracts caused moderate cytotoxicity (PAC/CE and FLO/SC). Nevertheless, because all extract treatments and V caused cell death, the cytotoxic effect observed is mostly associated with the concentration of ethanol and DMSO used in the vehicle. Therefore, the reduction of the concentration of ethanol and DMSO should be considered for further studies. Furthermore, the two treatments that were moderately cytotoxic (PAC/CE and FLO/SC) could have antimicrobial and antibiofilm activities because of the toxicity observed in the cytotoxicity assay. Also, the methodology of MTT has its limitations [85], because the oral mucosa is a tissue with three-dimensional organization and not monolayer cells, and therefore toxicity studies should be performed using in vivo models and even more than one assay should be used to determine cell viability in vitro studies, as this would increase the reliability of the results obtained. Moreover, a reduction in the cytotoxicity of these extracts could be achieved by isolating the active fraction from the crude extract, which should be better evaluated, and/or decreasing the concentration of EtOH and DMSO in the vehicle.

Caution is required in interpreting the data presented to avoid overestimation of the effects for the control of cariogenic biofilms. Here, simple models were used to verify which extracts have potential antimicrobial and/or antibiofilm activities (which usually employ an exposure time of 24 h during screenings [38]) and evaluated those that had an inhibitory outcome also for cytotoxic effect against oral keratinocytes organized in monolayers after 1 h exposure (which do not mimic the complex organization of the oral mucosa tissue). Moreover, to test further the effect of selected extracts with ‘promising’ antibiofilm activity, we used models for GtfB activity and *S. mutans* adhesion to treated pellicle with exposure time of 30 min, while for microbial adhesion to glucan matrix, the treatments were present for 4 h (which are the exposure times established in the literature) [11, 12, 39, 40]. As described above, the next step of this line of research, in addition to using optimized concentrations of the extracts, will be to evaluate the extracts and AcOEt fractions using more complex models with shorter exposure time to mimic a clinical application.

The content of flavonoids and diterpenes could be standardized in extracts and promising fractions, overcoming problems of compositional variation associated with phytochemical extraction and geographic or seasonal influences. It is noteworthy that because *C. sylvestris* flourishes and grows fruit in the second year of life [86] and lives at least 20 years [87], cultivation is favorable for therapeutic purposes. Also, its wood can be used as fuel and for the construction of fences, posts, stakes, rustic carpentry, and cable tools [87]. Therefore, the controlled cultivation of *C. sylvestris* may contribute to the development of new agents for the control of cariogenic biofilm, which reduces the final cost when compared to the purified compounds. Although this study used simplistic in vitro models, the results substantiate the ethnobotanical use of extracts and fractions of specific biomes and varieties for the prevention of oral cariogenic biofilms. However, in vitro data from more complex (e.g., microcosm) and in vivo models may be useful in determining the potential utility of this plant.

## Conclusions

Specific extracts and fractions of *C. sylvestris* are effective in inhibiting pathogenic microorganisms related to dental caries and are therefore promising candidates for the development of a dental caries preventive formulation.

## Data Availability

Data sets generated during and/or analysed during the current study are available from the corresponding authors upon reasonable request.

## Abbreviations

DMSO: Dimethyl sulfoxide
EtOH: Ethanol
IC_50_: the inhibitory concentration of the extract or fraction that inhibits growth of the microorganisms by 50%
TYE: Tryptone yeast extract
TYEg: Tryptone yeast extract with glucose
TYEs: Tryptone yeast extract with sucrse
V: Vehicle
var: variety
